# Quantification and characterization of microplastics (MPs) pollution in peri-uburban agricultural lands of Lahore, Pakistan

**DOI:** 10.1371/journal.pone.0291760

**Published:** 2023-10-03

**Authors:** Muhammad Luqman, Tehreem Shahid, Muhammad Umer Farooq Awan, Saif Ur Rehman Kashif, Fariha Arooj, Ali Raza Awan

**Affiliations:** 1 Department of Environmental Sciences, University of Veterinary & Animal Sciences, Lahore, Pakistan; 2 Department of Botany, Government College University, Lahore, Pakistan; 3 Institute of Biochemistry and Biotechnology, University of Veterinary & Animal Sciences, Lahore, Pakistan; VIT University, INDIA

## Abstract

Microplastics (MPs) contaminate every conceivable terrestrial and aquatic environment including high peaks and deep marine trenches. Agricultural lands alone are expected to receive plastic up to 23 times more than ocean basins. In this study, soil samples were collected from peri-urban agricultural lands of Lahore on four sides including Kala Shah Kaku (KSK), Punjab University (PU), Dera Gujran (DG), and Sagian (SG). National Oceanic and Atmospheric Administration (NOAA) protocol was used for MPs extraction and analysis. Extracted MPs were analyzed under microscope at 40X magnification and their composition was analyzed using Fourier Transform Infrared (FTIR) spectroscopy. A considerable concentration of MPs was recorded at all sites. The highest contamination was found at SG with 876 ±194 MPs/kg of soil, and the lowest contamination was recorded at PU with 672 ±235 MPs/kg of soil. However, these differences among the sites were not statistically significant (*p* = 0.29). The overall predominant shape of MPs was fibers (613±71, 79.73%) followed by sheets (125±55, 16.28%), fragments (30±5, 3.9%) and foam particles (1±2, .09%). The differences in the distribution of MPs in various types were statistically significant (*p* = 0), while differences between sites were insignificant (*p* = 0.13). About 95% of MPs were less than 2 mm and 85% were less than 1 mm size. The distribution of MPs in various sizes (*p* = 0) and differences of this distribution between sites (*p* = 0.037) were both statistically significant. A good diversity of nine colored MPs was recorded, however majority of the MPs were transparent (89.57%). Six polymer including Polyethylene (PE), Polyethylene terephthalate (PET), Polypropylene (PP), Polystyrene (PS), Polycarbonate (PC), and Polyvinyl Chloride (PVC) were identified by FTIR. The current levels of MPs pollution are higher than in many other parts of the world. Composition of MPs (types, colors, sizes, and polymer types) indicates the diversity of their sources and their possible implications on agricultural ecosystem.

## Introduction

Plastic production, its indiscriminate use and mismanagement of plastic waste are the burning environmental issues. When this durable polymer is exposed to temperature fluctuations, weathering, erosion and UV rays, it disintegrates into smaller components of < 5 mm in size, called microplastics (MPs) [1]. Any synthetic or semi-synthetic polymer with thermoset and thermoplastic characteristics is defined as plastic [2]. Plastics can be synthesized from hydrocarbon chains or biomass raw materials. Production of plastic has seen an exponential growth since its arrival on the consumer stage. Owing to being cheaper, durable, versatile and portable, the plastic have become an integral part of human life [3]. Today, our globe without plastic and polymers seems unrealistic and unimaginable. Plastic was not used outside of the military sector until the end of World War II but now plastic production has surpassed every type of man-made material in the world [4]. Plastics are polymers that can be reshaped and reused after heating. Many types of plastic are produced synthetically including nylon, polyester, polystyrene (PS), polyethylene (PE), polyvinyl chloride (PVC), Teflon, low- and high- density polyethylene (LDPE and HDPE), etc. The global plastic production in 2018 was close to 360 million tons, for which China (30%), Europe (17%), and North America (18%) primarily manufactured the raw materials [5].

A large amount of plastic waste is released into the environment due to mismanagement and mishandling of solid waste [6]. It reaches oceans, beaches, agricultural lands and water reservoirs making them hotspots of plastic pollution. The released plastic waste disintegrates into smaller size debris by UV rays, weathering and other chemical, physical and biological drivers [7]. Polymers are also degraded by photochemical reactions which include thermal, photo and mechanical degradation while hydrolysis and oxidation contribute to chemical and biological pathways [8]. Once disintegrated, they become macroplastics (<25–5 mm), MPs (5 mm-1 μm), nanoplastics (<1 μm), and some large particles [9]. MPs are released into the environment as primary or secondary MPs. MPs that are originally manufactured in microscopic size particles are primary MPs [10]. Secondary MPs are produced due to degradation, disintegration and fragmentation of large size plastics [11]. MPs types include fibers, films, fragments, foams and pellets which come from numerous sources manufactured and used in the environment [12].

MPs are exposed to a range of chemicals after releasing into the environment and cause direct as well as indirect toxicity to human beings, terrestrial and marine biota [13]. Direct toxicity is caused by stabilizers, dyes and different toxic additives/chemicals [14]. Common additives of plastics like phenols, and esters continue to spread in surroundings, leading to lasting and severe impacts on all life forms and the environment [15]. Chemicals like polychlorinated biphenyls (PCBs) and polycyclic aromatic hydrocarbons (PAHs) can attach to their surfaces causing combined toxicity [11]. Indirect toxicity is caused by the hydrophobic nature of microplastic and their surface area to volume ratio. Numerous chemicals are absorbed onto the surface of MPs and they react with the chemicals when exposed to sunlight, oxidation and other chemical processes, becoming an entirely new species and causing toxicity in a new way [16]. Common MPs like PE, PS, PP and also PVC can adsorb organic pollutants 3–6 times more in the presence of co-existing surfactants [17,18].

MPs pollution in agricultural soils is a recent challenge for environmental sciences and agriculturists as sources, release, transportation, fate and effects of MPs in terrestrial ecosystem are relatively little acknowledged compared to an aquatic environment [19]. MPs are likely to be present more abundantly in soils, where they are produced, compared to aquatic ecosystems. MPs in the soils may pose risks to food security, food quality and human health [20]. MPs can enter the agricultural soils through various means like use of plastic based biosolids, irrigation with wastewater and use of plastic mulches [21]. Complex nature of soil matrix can increase the aggregation level of MPs causing ambiguous threats to the crops [22]. Further, plastic tunnels for vegetables, plastic labels and protection covers of vegetables and fruits are noteworthy origins of input of MPs in agricultural soils [23]. Compost, produced by biodegradable waste material, is used as fertilizer in every part of the world. Inappropriate waste separation can lead to a large fraction of plastics and MPs (when disintegrated) into the soil [24]. Soils act as long-lasting sink to accumulate MPs leading to affecting the agroecosystems even more adversely. In addition, different farming practices like ploughing, irrigation, and fertilization lead to horizontal and vertical movement of MPs in soil [25]. These MPs can further reach to water channels via agricultural runoffs ending up in aquatic environments [26].

MPs in agricultural soils affect plant communities directly, as increasing fragmentation leads to a decrease in size of MPs, through accumulation of various sizes and shapes in root, stem, and leave [27]. Furthermore, MPs can reduce the uptake of water and affect the nutritional status by blocking seed pores. Indirect impacts of MPs include the varying physiochemical attributes of the soil and soil-dwelling microbes [28]. Introduction of MPs into soil leads to increased soil water evaporation and desiccation of surfaces which further affect vertical uptake of water [29]. Phytotoxicity, and significant reduction in maize biomass and chlorophyll content in leaves have been observed due to soil contamination with Polylactic Acid (PLA), which is thermoplastic polyester [30]. MPs with heavy metals can drive changes in plant performance [30]. Edible plants are directly subjected to MPs when manure, bio fertilizers and plastic mulches are applied. MPs can pose a serious threat to crops as they may block seed pores and can limit the uptake of nutrients and water from roots [28]. They can also impact soil organisms like earth worms and are vertically moved through their feces [28]. Presence of cadmium (Cd) with a high concentration of polylactic acid (PLA) may cause a strong phototoxicity [30]. Coexistence of MPs and Cd can jointly drive shifts in plant performance and root symbiosis, thereby posing additional risks for agro-ecosystems and soil biodiversity [30,31].

Lahore is a capital city of the Province of Punjab, Pakistan with about 14 million population [32]. Non availability of canal water, high costs of electricity and fertilizers, and lack of regulations convince the farmer community to use untreated wastewater (purely or mixed with irrigation water) for growing vegetables and crops in peri-urban agricultural lands of Lahore [33]. The use of wastewater (containing domestic sewage and effluents from hundreds of textile and other industries) has the potential to bring lots of MPs in such agricultural lands. The use of plastic tunnels to grow vegetables and dumping of domestic and construction waste near these lands further aggravate the situation. These practices are posing serious risk of microplastic (MPs) pollution in agricultural soils and threat to agricultural ecosystem and human health. The status of MPs pollution in these agricultural lands was direly needed. Under these circumstances, this study was conducted to assess the magnitude of MPs pollution in peri-urban agricultural lands of the Lahore, Pakistan.

## Materials and methods

### Sampling sites

**Fig 1:**
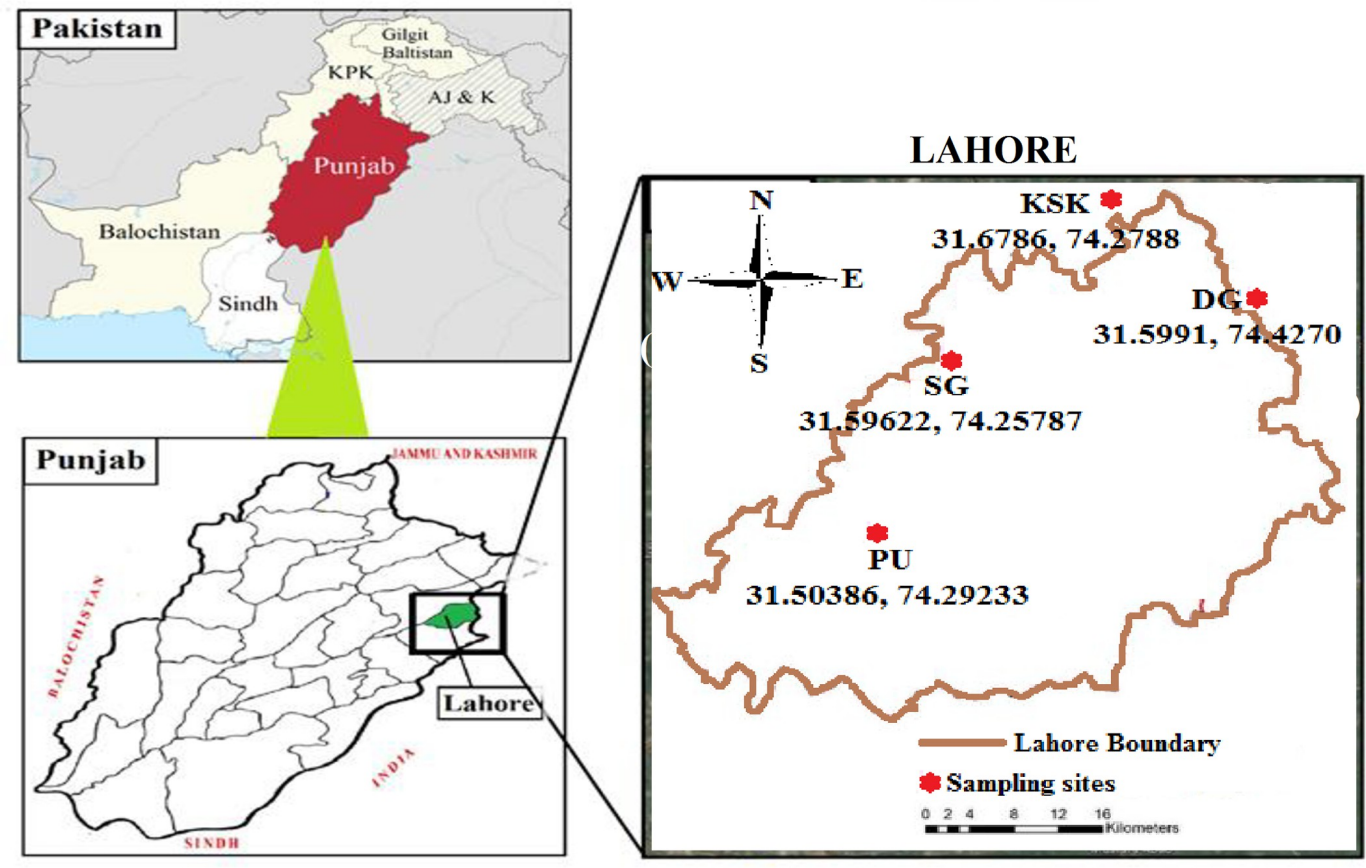
Sampling sites for MPs analysis in peri-urban agricultural lands of Lahore, Pakistan.

Lahore, the second largest city of Pakistan, is a business, residential and industrial hub of the country with an area of 1,772 km^2^. It receives a large amount of plastic waste. In this study, four peri-urban agricultural lands in the vicinity of Lahore were selected to quantify the level of MPs pollution in soil. One acre from each sampling site (northern, southern, eastern and western side of the city) was selected for sampling (Fig 1). Soil sampling for research from peri-urban agricultural lands of Lahore required no permit from any city or provincial authority, hence no permit was obtained. Agricultural lands including Punjab University (PU), Sagian (SG), Kala Shah Kaku (KSK), and Dera Gujran (DG) are neighbouring residential areas of Lahore city.

Punjab University (PU) site is situated in about 8 km^2^ pocket of agricultural land touching the western border of the Lahore city. This pocket is dedicated to the University of the Punjab’s new campus. Wheat and fodder are the main crops grown in this pocket of agricultural land. Vegetables are also grown on some acres. Use of plastic tunnels to grow vegetable has not been observed in this piece of land during winter season. There is no nearby slum to this land except University Faculty residential area and properly managed societies which are always much cleaner than slums. Impact of improper disposal of plastic bags and waste from nearby population is relatively less. This site is one kilometer away from Wahdat Road (Light traffic, with high intensity) and Maulana Shaukat Ali Road (Light traffic with high intensity), and 1.25 kilometer from Canal Road (Light traffic with high intensity).

Sagian site is a pocketed agricultural land in outer urban sprawl, about seven KM west of Lahore city’s outer perimeter. This site is 350 meter away from M2 Motorway (Heavy and light trafiic with high intensity) and about 100 meter from Lahore-Jaranwala road (light traffic with low intensity). Main crops include fodder and vegetables with continous use of low volume of domestic sewage and industrial wastewater. Use of plastic tunnels in winter to grow vegetable has also been seen frequently on this site. Compost ferlizers are also used at this site. Significant impact of improper dumping of domestic waste (including plastic waste) is evident on this site due to nearby low-income population in unplanned slums.

KSK site is seven kilometer in north of lahore city’s outer perimeter. Two major roads including M-11 motorway (light traffic, moderate intensity) and N5-GT road (light and heavy traffic, high intensity) are about 2.5 kilometer and 2.7 kilometer away from this site respectively. Two other roads also pass more than 2.5 KM away from this site i.e. Kalakhatai road (light traffic, low intensity) and Lahore bypass road (Light traffic, low intensity). Wheat, rice, and fodder are main crops on this site, while vegetable are also frequently grown. Tunnel farming is used to grow the non-seasonal vegetables in winters. Use of the compost ferlizers is a usual phenomenon at this site. Occasionally, to reduce input costs, farmers use untreated wastewater for growing vegetables. Impact of improper dumping of domestic waste is common from residents in nearby settlements.

Dera Gujran (Site) is 3.5 KM in the east of Lahore city’s outer perimeter. This site is one kilometer from the Lahore ring road, which has light traffic of low intensity. Rice and wheat are the main crops in these lands, however pockets of fodder are also frequently grown. Lakhoder landfill site is two kilometer east of this sampling site.

### Sample collection

Samples were collected on clear sunny days of winter season in the month of December 2021. Five replicate soil samples were collected from each sampling site randomly (one sample from each corner of the acre and one from the centre). The total number of samples taken for the study was twenty (n = 20). Samples were collected using a quadrant of one square foot by placing it randomly at each sampling site and upper 10 cm of soil layer was taken as sample with the help of a spade. Soil samples were taken in aluminum bags and transported to the laboratory for further analysis.

### Sample preparation and density separations

*MPs* were extracted by following the standard protocol of National Oceanic and Atmospheric Administration (NOAA) for MPs extraction from sediments [34]. Samples were sun-dried through the windowpane in the laboratory in trays covered with aluminum foil, for 24 hours, to avoid plastic contamination and stored in glass jars. Samples were initially sieved with a sieve of 5 mm mesh size to remove all the particles greater than 5 mm. Soil of 200 g from each sample was added into a 1000 mL glass beaker and dried overnight in the oven at 80°C to dry completely. Samples were then subjected to density separation to extract floating materials by adding 500 mL of supersaturated NaCl (5 M) solution (density = 1.15 g/mL) to the beaker of dried sample. Mixture was vigorously shaken with the help of a metal spatula for three minutes to float out MPs and beaker was kept on rest for an hours. Then floating solids were sieved through 0.06 mm mesh size customized sieve. Walls of the beaker were rinsed carefully with 5 M NaCl solution and all the residual solids were poured into the sieve. The procedure was repeated five times to extract all the floating material on the sieve, while the settled solids were discarded in the end. Debris collected over the 0.06 mm sieve was put in the glass beaker and oven dried at 80° C for 24 hours or longer until dryness. Although, the supersaturated NaCl solution have some limitations to separate out few high density polymers, yet most commonly used chemical in majority of protocoals including NOAA.

### Digestion by wet peroxide (WPO)

Digestion by WPO method was done following the standard protocol of digestion described by NOAA for MPs extraction from sediments [34]. Dried matter was subjected to the process of wet peroxide digestion by 0.05 M Fe (II) solution and 30% hydrogen peroxide (H_2_O_2_). Iron salt used in this process was FeSO_4_.7H_2_O. Twenty milliliters of 0.05 M Fe (II) solution was added to the beaker of dried solids, followed by 20 mL of 30% H_2_O_2_. Mixture was left in the laboratory at room temperature for five minutes and then heated on a hotplate with magnetic stirrer at 75°C until the bubbles appeared on the surface. Then beaker was placed in fume hood until “effervescence” was settled. Distilled water was added to slow down the reaction in case of overflow. Process of heating and addition of more H_2_O_2_ was done until all the organic solids were invisible and mixture became transparent. After completion of oxidation process, six grams of NaCl per 20 mL of sample was added to make the aqueous solution denser and mixture was heated to 75°C to dissolve the salt.

### Density separation

For density separation, WPO solution was transferred to density separator made with funnel and clamped rubber tubing. Beaker was rinsed carefully with distilled water to transfer all remaining debris to the funnel, which was then loosely covered with aluminum foil for overnight stay. Settled solids were visually inspected to see if any visible plastic particle was present. In case of visible MPs, particles were separated with forceps and the remaining material was drained. Floating particles were collected in a custom 0.06 mm clean sieve and the density separator was rinsed several times with distilled water. The sieve was air dried for 24 hours while loosely covered with aluminium foil to avoid contamination.

### Quality control

During sampling, transportation, drying, sieving, density separation and microscopic analysis, all the potential hotspots of cross contamination and particle loss were taken care to minimize the issue. No plastic container or equipment is used during the whole process. Distilled water used in the laboratory and all the reagents were filtered through 11 μm filter. All the glassware and apparatus used in MPs analysis was washed with filtered distilled water and kept covered with aluminium foil. A well cleaned part of the laoratory was dedicated to MPs research and no one was allowed in that area with any plastic and synthetic fiber bearing attire, cap, hair accessories, cosmetic use, googles, etc. Further, a procedural blank sample (wet filter paper) was parallel run with each sampling site to find the numbers of false positive particles from the environment. Number and types of MPs found in the blank were deducted from the total MPs count of each replicate. Total four procedural blanks (quadruplicate) were run along the samples. Blank sample (wet filter paper) sealed in aluminium bag were tranported to the sampling sites, exposed in the same environment in the field and put back into the aluminium and transported to the lab along-with samples. Representativeness of samples at each site was ensured by taking five replicates. Blank wet filter paper was kept with the samples in a beaker in the same laboratory environment till the digestion step. Then it was rinsed well in the same beaker and analysed for any MPs contents paralel with samples.

### Microscopic examination and FTIR analysis

MPs analysis was done at 40X magnification under microscope to identify the shapes, colors, and sizes of MPs on the sieve. Larger MPs (>2.0 mm), in visible range with naked eye, were picked from sieve with the help of forceps and transferred to clean glass vial for composition analysis of polymer types by Fourier Transform Infrared Spectrometry (FTIR). The compopsition of polymer type was determined by acquiring spectra using Therrmo Scientific Nicolet 6700 FTIR within the wavelength range of 4000–500 cm^-1^ with 8 sample scans and 8 background scans per spectrum, and resolution of 4 cm^-1^. Smple spectra were analyzed with Thermo OMNIC software and compared with OMNIC polymer spectral library for identification of polymer type. Minimum 70% recognition limit was set for polymer type identification.

## Results

During the current study, average abundance of MPs recorded was 672 (±235), 876 (±194), 831 (±220), and 699 (±54) MPs/kg of soils at Punjab University, Sagian, Kala Shah Kaku, and Dera Gujran sites respectively (Table 1). Highest contaminated site was Sagian (876 ±194), while the lowest was Punjab University (672 ±235). Most contaminated individual replicates were belonging to Sagian and Kala Shah Kaku (Sagian R-5, KSK-R-2), while least contaminated replicate were Punjab University R-1 and R-2. The observed differences in abundance of MPs among the four sites were not statistically significant (p-value 0.29).

**Table 1:** Abundance of MPs (number/kg of soil) in peri-urban agricultural lands of Lahore.

Replicates	Punjab University		Sagian	Kala Shah Kaku	Dera Gujran
**R-1**	390		705	600	765
**R-2**	495		825	1140	645
**R-3**	735		705	720	660
**R-4**	990		990	720	675
**R-5**	750		1155	975	750
**Mean (±SD)**	672 (±235)		876 (±194)	831 (±220)	699 (±54)
**SE**	105		87	99	25
**Median**	735		825	720	675
**ANOVA**					
**Source**	**DF**	**SS**	**MS**	**F-Stat**	**P-value**
**Between Groups**	3	148005	49335	1.3606	0.2904
**Within Groups**	16	580140.0295	36258.7518		
**Total**	19	728145.0295	19		

SD-Standard Deviation, SE-Standard error, DF-degree of freedom, SS-Sum of squares, MS-Mean square.

**Fig 2:**
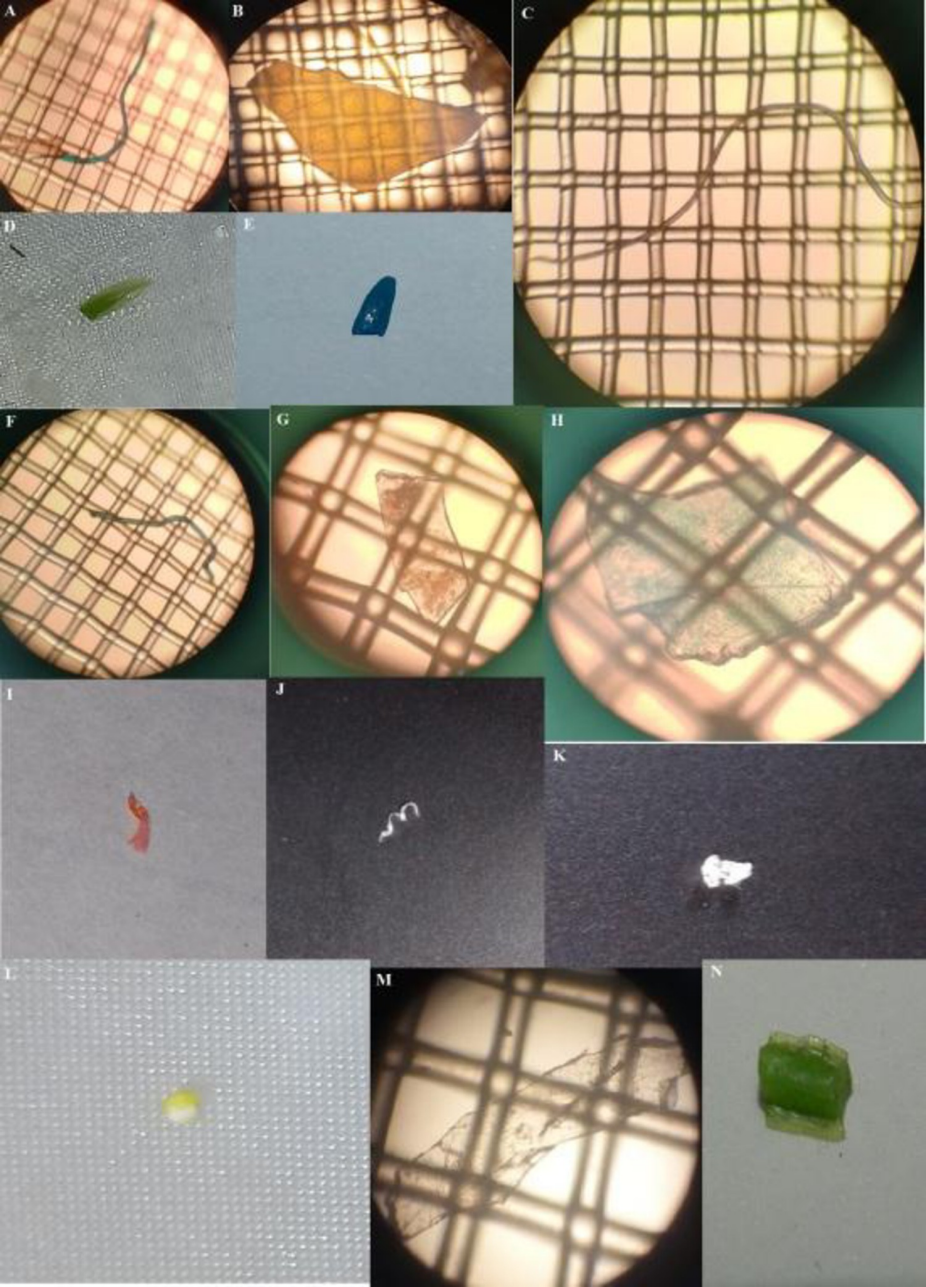
Images of some MPs in peri-urban agricultural lands of Lahore, Pakistan; A) fiber, B) film, C) fiber, D) fragment, E) fragment, F) fiber, G) sheet, H) sheet, I) fragment, J) fiber, K) fragment, L) Foam palette, M) sheet, N) fragment.

**Fig 3:**
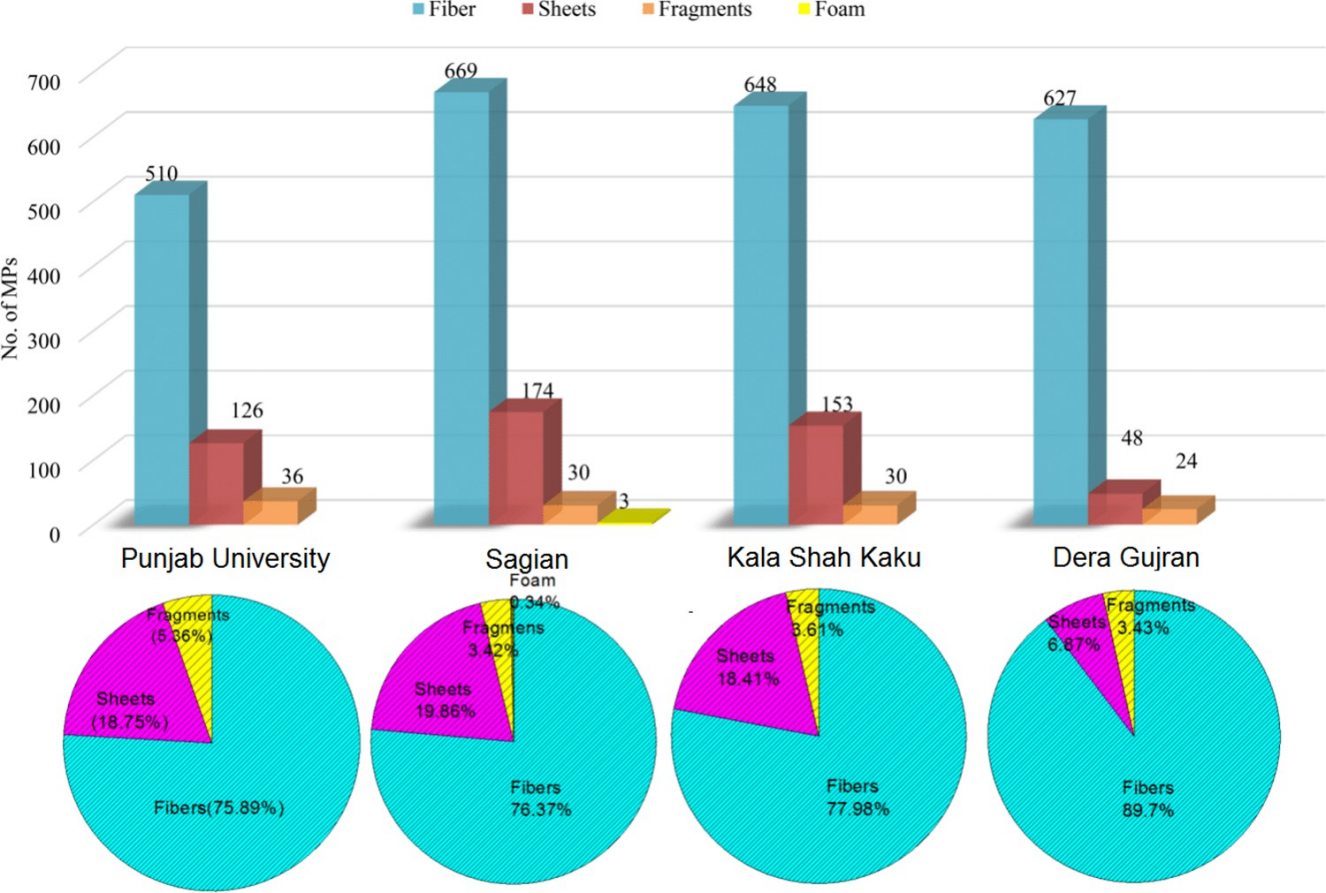
Average number of various types of MPs/kg of soil at each sampling site in peri-urban agricultural lands of Lahore, Pakistan.

**Fig 4:**
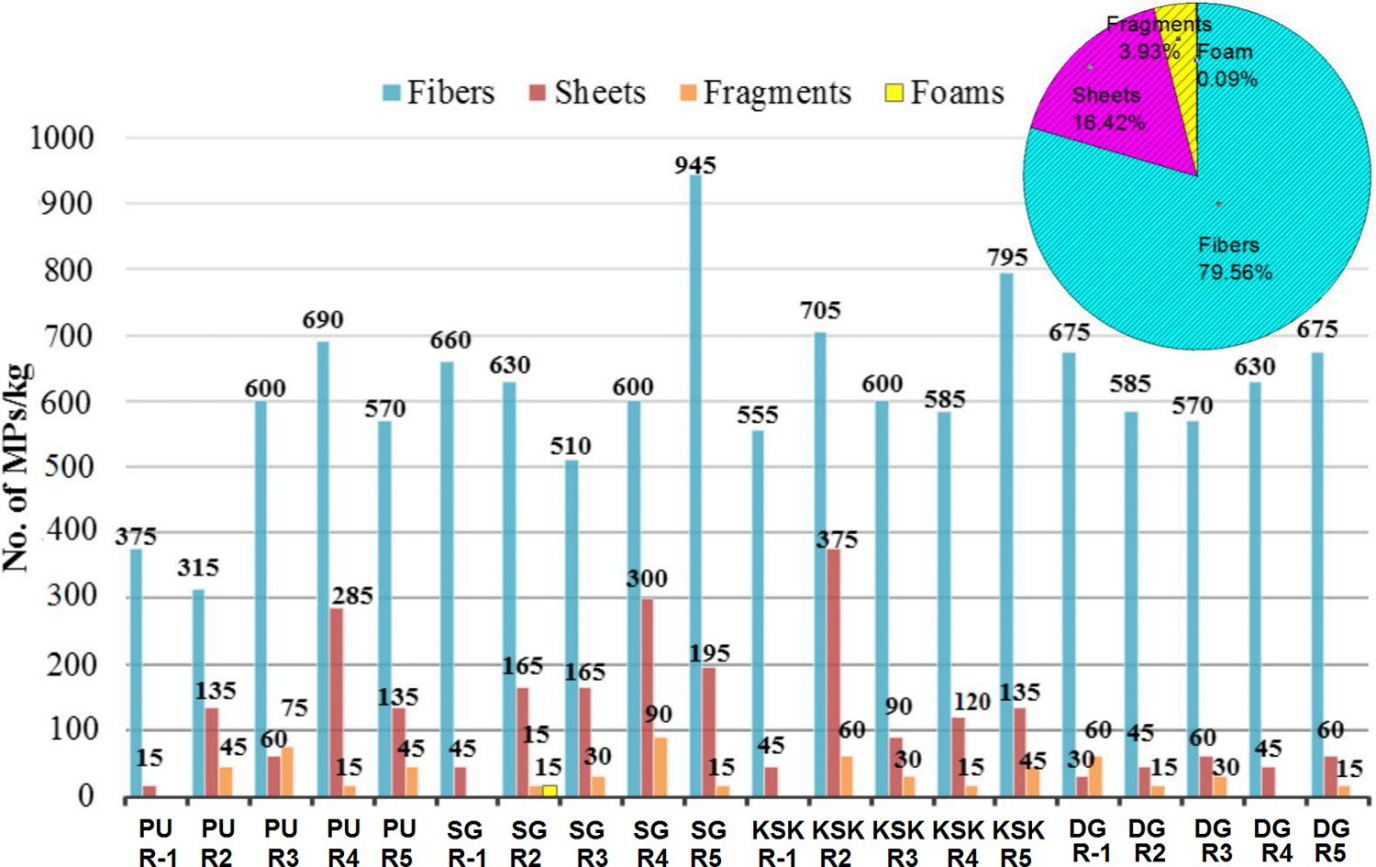
Concentration profile of various types of MPs/kg of soil in each replicate at all sites in peri-urban agricultural lands of Lahore, Pakistan.

**Fig 5:**
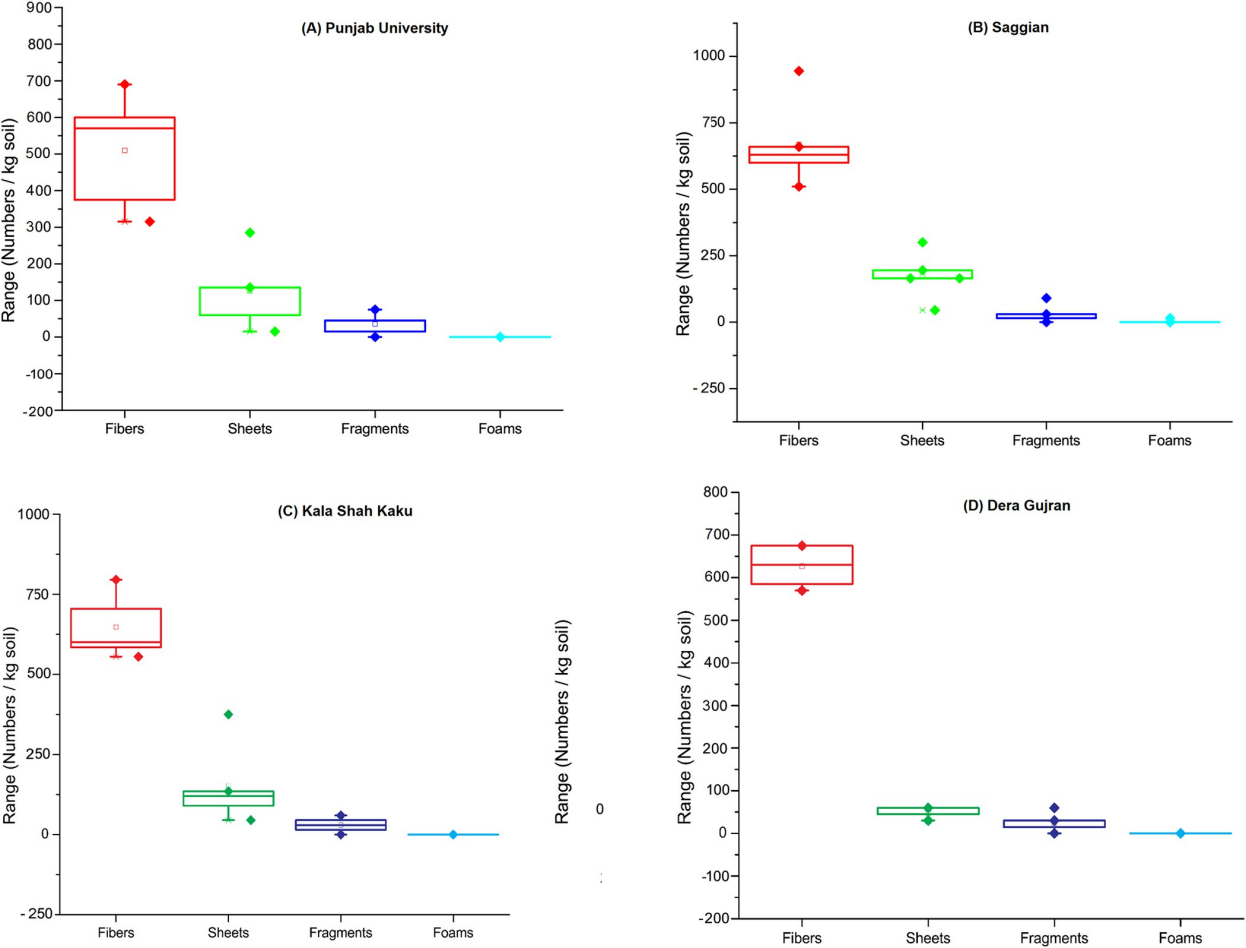
Box graph of types of MPs at each sampling site in peri-urban agricultural lands of Lahore, Pakistan.

Various types recorded during the current study included fibers, sheets, fragments and foam (Fig 2). Fibers were dominant at all sites ranging from 76–90% of the recorded types (Fig 3). Second most frequent type of MPs was sheets (7–20%), while third dominant type was fragments (3–9%). Foam particles were found very few in number (0.34%) and only at Sagian site. Collectively, including all the MPs at all replicates from four sampling sites, 79.56% were fibers, 16.42% were sheets, 3.93% were fragments, and only 0.09% of them were foam particles (Fig 4). Highest numbers of fiber were found at Sagian, R-5 with 945 fiber/kg of soil, while minimum numbers were at Punjab University R-2 with 315 fiber plastic per kilogram of soil. Sheets were next to fibers, and then fragments, while foam particles were recorded only in one replicate at Sagian R-2 with 15 numbers per kilogram of soil. Mean values of MPs types varied across all the four sites (Table 2, Fig 5). The differences in the distribution of MPs in various types at the four sites were statistically significant (p-value of 0.0) (Table 2). However, the difference in distribution of various types across the four sites is not statistically significant (p-value 0.13). Also the difference between the sample averages of all groups is not big enough to be statistically significant (p-value 0.19).

**Table 2:** Abundance of various MPs types (mean values, No./kg) found at all the four sites and their variance among the sites and types.

Sites/Types			Fibers	Sheets	Fragments	Foam
Punjab University			510	126	36	0
Sagian			669	174	30	3
Kala Shah Kaku			648	153	30	0
Dera Gujran			627	48	24	0
**Descriptive**	Mean		613.5	125.25	30	0.75
	SD		71.09852	55.12032	4.89898	1.5
	SE of Mean		35.54926	27.56016	2.44949	0.75
**Two-Way ANOVA**	**Source**	**DF**	**SS**	**MS**	**F Stat**	**P-value**
	Factor A—rows (A)	3	37001.25	12333.75	1.9129 (3,64)	0.1364
	Factor B—columns (B)	3	4898756.25	1632918.75	253.2577 (3,64)	0
	Interaction AB	9	84791.25	9421.25	1.4612 (9,64)	0.1818
	Error	64	412650	6447.6563		
	**Total**	**79**	**5433198.75**	**68774.6677**		

SD-Standard Deviation, SE-Standard error, DF-degree of freedom, SS-Sum of squares, MS-Mean square.

**Fig 6:**
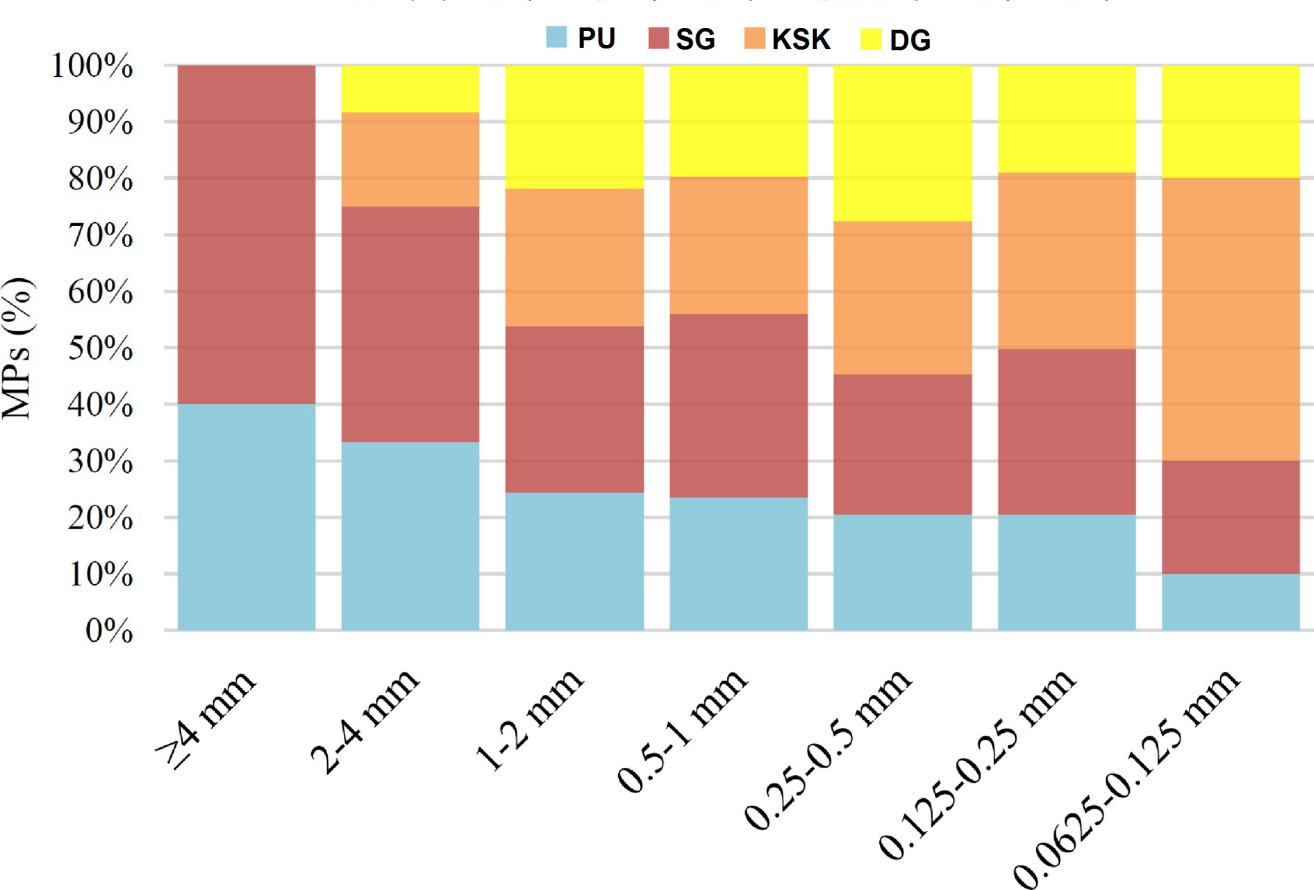
Percentage size fractions of MPs/Kg found at each site in peri-urban agricultural lands of Lahore, Pakistan.

**Fig 7:**
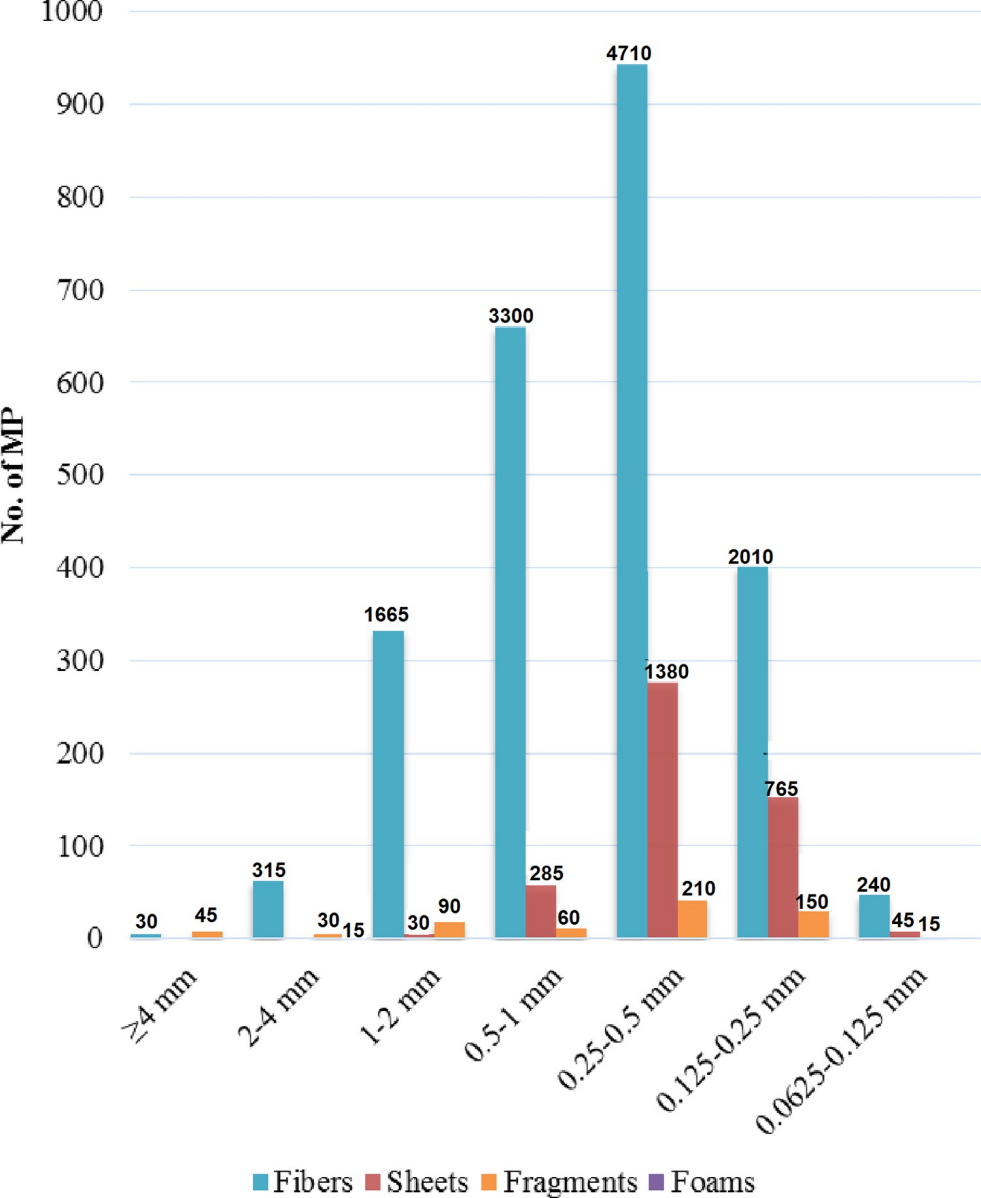
Size fractions of all MPs collectively found in peri-urban agricultural lands of Lahore, Pakistan.

Majority of the MPs found in peri-urban lands of Lahore were smaller in size. About 95% of MPs were less than 2 mm size and 85% were less than 1 mm (Table 3). About 41% of MPs were in the size range of 0.25–0.50 mm. Only 2.8% of MPs were found in the size range of 2–5 mm. Smallest size in the current study was 0.0625–0.125 mm and only 1.9% of the MPs were found in this range. Larger sized MPs (≥ 4 mm) uniquely were not recorded at Sagian and Kala Shah Kaku sites at all (Fig 6). Fibers were generally present in all size fractions, maximum being in size range of 0.25–0.5 mm (Fig 7). Next most occurring shape in all the size ranges was sheet, maximum being in size range of 0.25–0.5 mm. Foam particles were only observed in size range of 2–4 mm and were only 15 in number (Fig 7). The differences in the distribution of MPs in various sizes were statistically significant (p-value of 0.0) (Table 4). The differences in size distribution of MPs across the four sites were also statistically significant (p-value 0.036).

**Table 3:** Number of MPs with sizes and colors found in all samples at all sites collectively.

Size (mm)	Transparent	Green	Red	White	Blue	Purple	Brown	Black	Yellow	Total	Proportion	%age
**>4**	30	15	0	30	0	0	0	0	0	75	0.5	2.8
**2–4**	210	0	60	15	15	30	0	0	30	360	2.3	
**1–2**	1455	30	150	75	15	30	0	15	15	1785	11.6	95.2
**0.5–1**	3195	90	255	30	15	60	0	0	0	3645	23.7	
**0.25–0.5**	5895	120	150	15	60	0	60	0	0	6300	40.9	
**0.125–0.25**	2700	90	60	30	45	0	0	0	0	2925	19	
**0.0625–0.125**	300	0	0	0	0	0	0	0	0	300	1.9	1.9
**Total**	13785	345	675	195	150	120	60	15	45	15390	100	100

**Table 4:** Distribution of MPs in various size fractions at all sites and two-way analysis of variance.

Sites/Size range			0.0625–0.125 mm (No./kg)	0.125–0.25 mm (No./kg)	0.25–0.5 mm (No./kg)	0.5–1 mm (No./kg)	1–2 mm (No./kg)	2–4 mm (No./kg)	≥4 mm (No./kg)
Punjab University			**30**	**600**	**1290**	**855**	**435**	**120**	**30**
Sagian			**60**	**855**	**1560**	**1185**	**525**	**150**	**45**
Kala Shah Kaku			**150**	**915**	**1710**	**885**	**435**	**60**	**0**
Dera Gujran			**60**	**555**	**1740**	**720**	**390**	**30**	**0**
**Descriptive**	Mean		75	731.25	1575	911.25	446.25	90	19.75
	SD		51.96	180.16	205.67	196.10	56.62	54.77	22.5
	SE of Mean		25.98	90.08	102.84	98.05	28.31	27.39	11.25
**Two-Way ANOVA**	**Source**	**DF**	**SS**	**MS**		**F Statistic**		**P-value**	
	Factor A—rows (A)	3	21143.5714	7047.8571		2.9304 (3,112)		0.03672	
	Factor B—columns (B)	6	1555354.286	259225.7143		107.7822 (6,112)		0	
	Interaction AB	18	52431.4286	2912.8571		1.2111 (18,112)		0.2645	
	Error	112	269370	2405.0893					
	**Total**	**139**	**1898299.286**	**13656.8294**					

SD-Standard Deviation, SE-Standard error, DF-degree of freedom, SS-Sum of squares, MS-Mean square.

**Fig 8:**
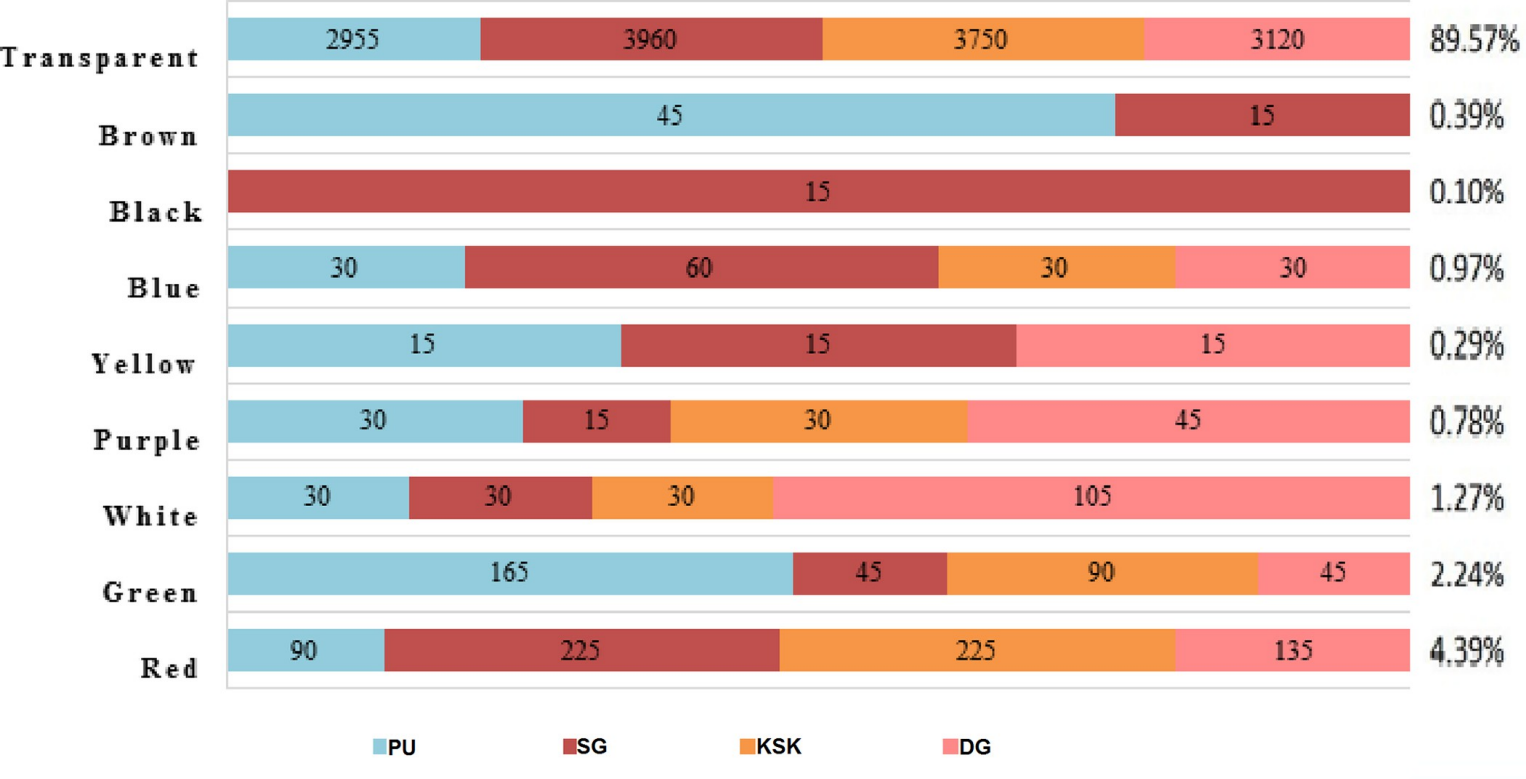
Color variation of total MPs found at all sites collectively in peri-urban agricultural lands of Lahore, Pakistan.

In current study, MPs were observed in nine different colors including red, green, white, purple, yellow, blue, black, brown and transparent (Table 3, Fig 8). Majority of them (about 90%) were transparent consisting of fibers, sheets and fragments. The largest fraction of transparent MPs was reported in Sagian soil samples. Further, the black color MPs were only observed at Sagian site.

MPs in the size range of 1–2 mm have shown highest color diversity with green, red, white, blue, purple, black and yellow MPs (Table 2). Only transparent MPs were found in the smallest size range fraction of 0.0625–0.125 mm, however, next size range from 0.125–0.25 mm, in addition to transparent particles, green, red, blue and white MPs were recorded. Other colors including blue, purple, brown, black, and yellow were absent altogether in size from 0.0625–0.25. Larger size fraction has shown greater color diversity compared to lower size.

**Fig 9:**
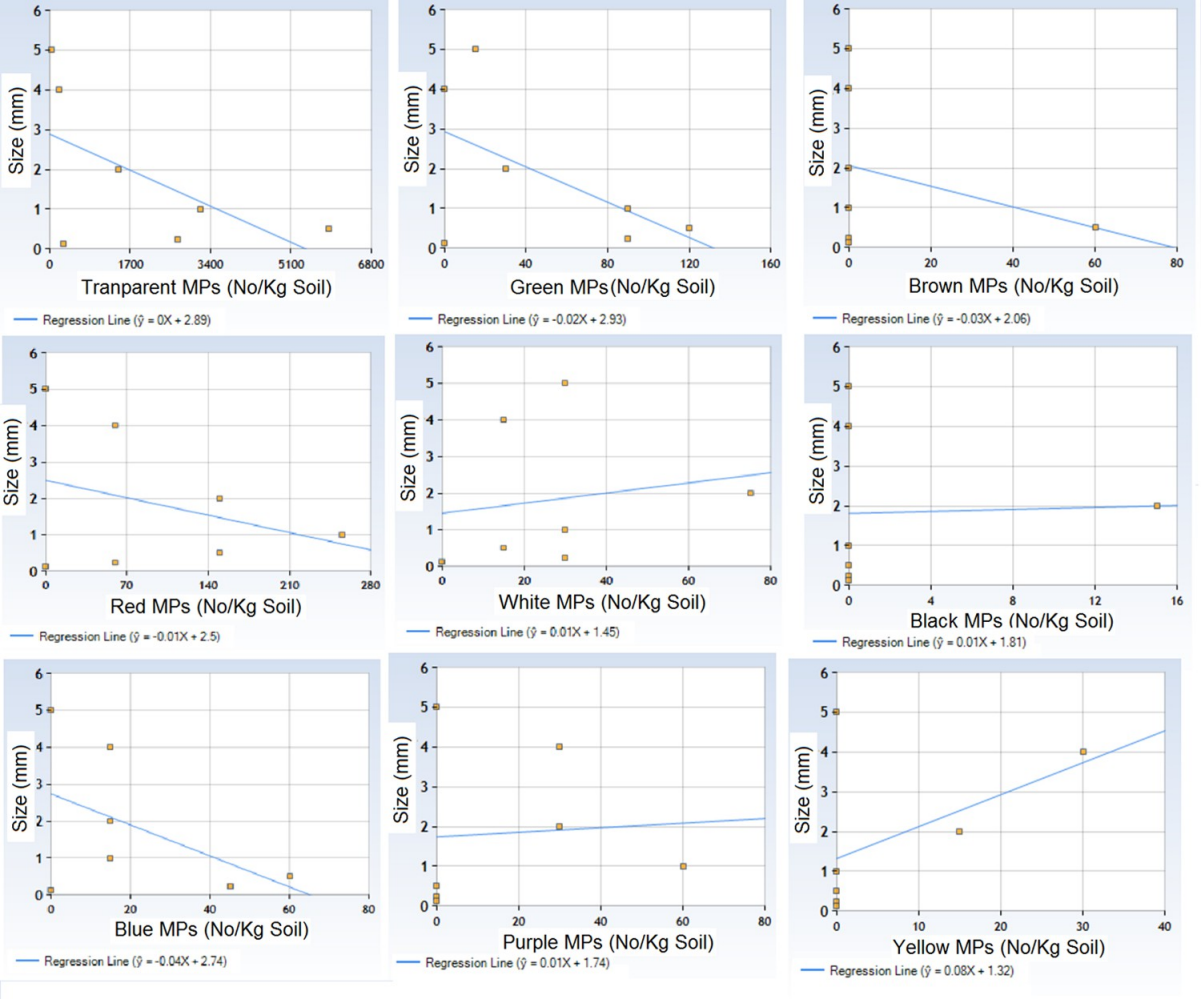
Correlation between size and abundance of various coloured MPs (Reqression equations) in peri-urban agricultural lands of Lahore, Pakistan.

Regression equations show that there was a relationship between numbers of transparent, green, brown, red, and blue color MPs with their size (Fig 9). Their number increases with decrease in size and vice versa. However, such relationship was not observed for white, black, purple and yellow.

**Fig 10:**
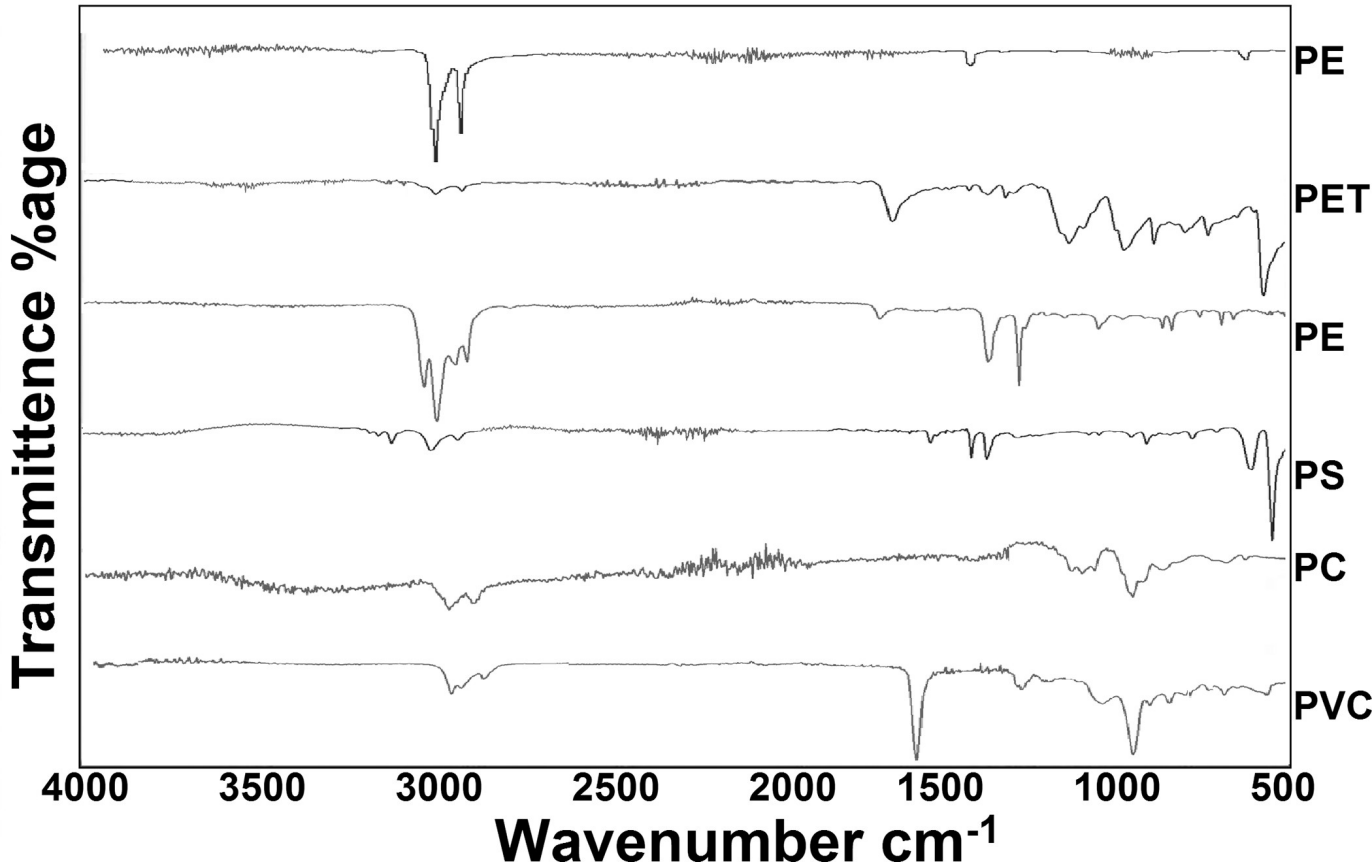
Polymer types of MPs found in peri-urban agricultural lands of Lahore, Pakistan.

**Fig 11:**
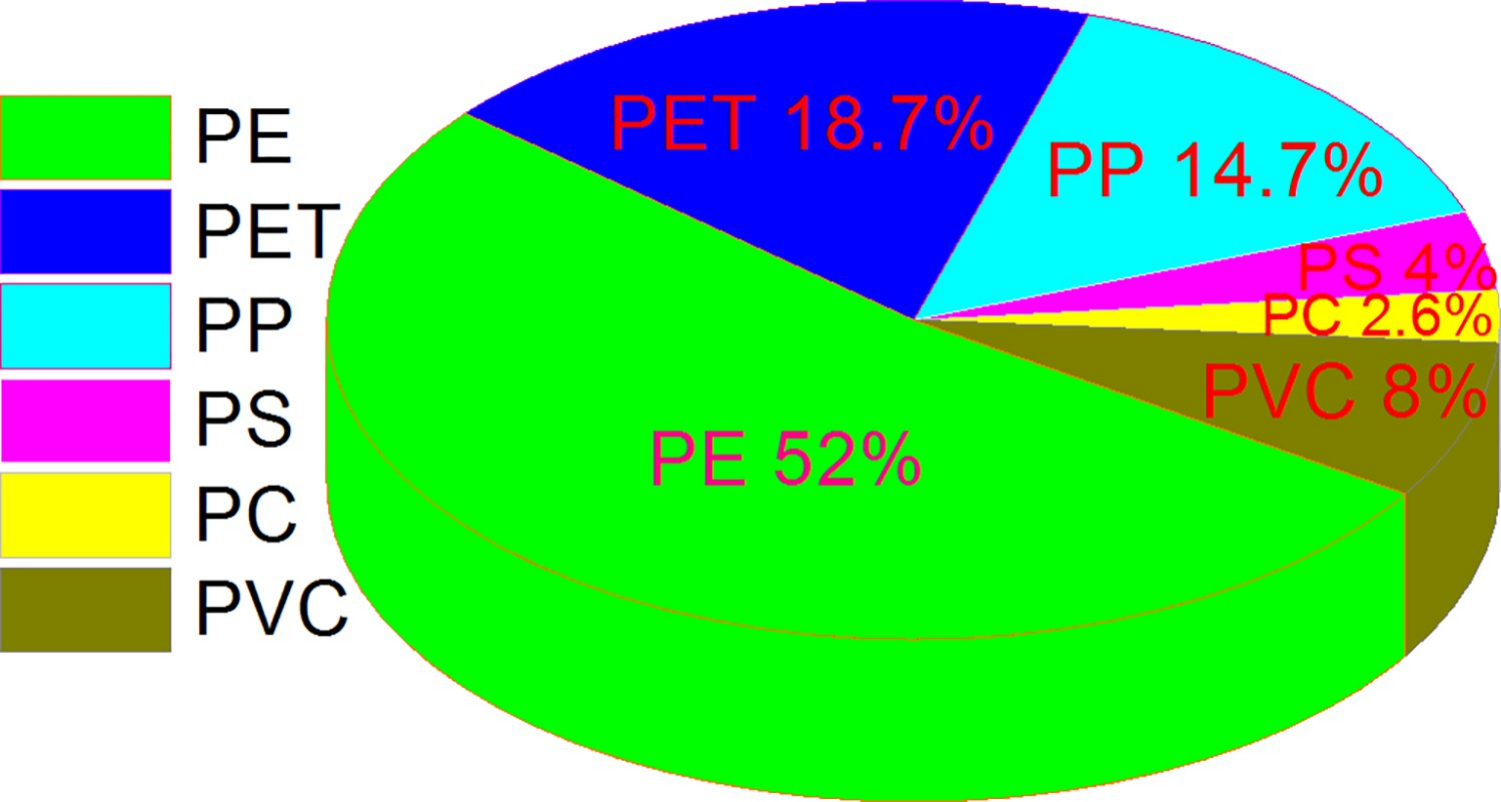
Proportion of various Polymer types in fragments of < 2 mm size in peri-urban agricultural lands of Lahore, Pakistan.

Six types of plastic polymers are recorded in the current study including Polyethylene (PE), Polyethylene terephthalate (PET), Polypropylene (PP), Polystyrene (PS), Polycarbonate (PC), and Polyvinyl Chloride (PVC) (Fig 10). Total 75 fragments of more than 2 mm size (>2–4 mm = 30 MPs, ≥ 4 mm = 45 MPs) were picked up for FTIR analysis. Among these, 16, 22, 24, and 13 particles were from PU, SG, KSK, and DG sites respectively. Of the 75 particles analysed by FTIR, 39 (52%) were PE, 14 (18.6%) PET, 11 (14.6%) PP, 3 (4%) PS, 2 PC (2.6%), and 6 (8%) PVC (Fig 11).

## Discussion

Abundance of MPs in agricultural soils recorded during the current study was higher than reported in many countries including Mauritius, Hungary, Chile, Germany, Japan, and Thailand, yet lower than many others (Table 5). Although the observed differences in abundance of MPs among the four sites were not statistically significant, PU and DG sites were apparently relatively cleaner with less MPs concentration in the soil compared to SG and KSK. Factors including use of plastic tunnels, plastic mulches, compost fertilizer, little or no wastewater were lacking at these two sites compared to SG and KSK where these factors were conspicuous. Also at PU and DG sites, no vegetables were grown in contrast to SG and KSK. For vegetables, the use of wastewater, tunnels and mulches is common in Pakistan compared to major crops like wheat, rice and fodder. Further near PU site no slums and dumping sites were spotted. Although DG site was only 2km from a city’s dumping site, yet relatively low MPs were recorded probably due the lack of earlier mentioned factors for SG and KSK. At SG and KSK sites there were slums and dumping sites for garbage including plastic. SG and KSK have nearby roads with heavy traffic compared to PU and DG that have light traffic roads near them. SG, which is most contaminated site has motorway with intense heavy and light traffic just 350 meters away.

**Table 5:** Comparison of MPs concentration in peri-urban agricultural lands of Lahore, Pakistan with global research.

Country	Abundance (MPs/kg)	Polymer types	Reference
**Tanzania**	Up to 1500	PET, HDLE, LPE, PS, etc.	[35]
**Mauritius**	Up to 420.0 ± 244.0	PP and PA	[36]
**Tunisia**	Up to 852.24 ± 124.2	PEVA, PE, PBAT, and PP	[37]
**Chile**	Up to 306	PE, PP, and PS	[38]
**Canada**	Up to 4144	PE, PP, PS, etc.	[39]
**Hungary**	Up to 225 ± 61.69	PE, PVC, and PP	[40]
**China**	Up to 8885	PE, PP, and PET	[41]
**China**	Up to 754 ± 477	PP, PE, PET, and PES	[42]
**China**	Up to 149.2 ± 52.5	PES, PP, and PS-AC	[43]
**Korea**	664	PE and PP	[44]
**Greece**	301 ± 140	PE and BMF	[45]
**Spain & Netherland**	2242 ± 984	NA	[46]
**Chile**	360	PE, PP, and PS	[47]
**Germany**	14.6	PES, PA, PVC	[48]
**Japan**	144	PE	[49]
**Switzerland**	10.5 ± 9.5	-	[50]
**Thailand**	12–117	PE, LDPE, PP, and PS	[51]
**Pakistan**	672 (±235) to 876 (±194)	PE, PET, PP, PS, PC, PVC	Current study

In current study, four types of MPs including fibers, sheets, fragments and foam were recorded in the soil samples of peri-urban agricultural lands of Lahore. Fiber was the most abundant type of MPs found following sheets, fragments and foams. Fibers were 79.73%, while sheets, fragments and foams were 16.28, 3.9 and 0.09% respectively. This relative abundance of MPs types is similar to the results reported in the shallow soils of Shanghai, China, where fibers were found in highest percentage (53.33%) followed by fragments (37.58%), films (6.67%) and pelettes (2.12%) [52]. Similar results were recorded by Zhang and Liu in 2018 [53] in agricultural lands of south-western China, where fibers were recorded up to 92.1% of all MPs found while little concentration of fragments (4.1%) and films (3.7%). Supporting results were also recorded by several other studies while exploring MPs concentration in agricultural lands [54–56]. Greater concentration of MP fibers in the current study is suspected to be contributed by city dust from Lahore. Further reason could be the use of sewage water mixed with industrial effluents especially from textile industries especially at SG and KSK sites. Similarly MP fibers could be significantly contributed from domestic sewage where laundries are producing lots of fibers. Similarly polyester, acrylic, nylon, and several other plastic fibers used in winter clothes do also contribute to city dust and ultimately to this fraction of MPs in peri-urban agricultural lands of Lahore. According to Lahore Chamber of Commerce and Industry (LCCI), there are about 984 textile industries registered with LCCI [57]. Smaller hosiery and yarn setups are apart from these registered large textile setups. Textile industry seems to be one of the major contributors to the fiber pollution and they can reach agro-ecosystems through air, water, waste and other technologies using on the site. The second abundant type of MPs was sheets or film, which might have come from the agricultural practices like plastic mulching, plastic tunnels, and dumping of waste including plastic especially at SG, KSK sites. Possible sources of fragments could also be the waste material consisting of a range of plastic utensils, personal products, pet bottles, etc. in the dumping sites. The distribution of MP types across the four sites was not statistically significant despite the apparent presence of all of the types at SG and KSK sites in greater numbers. However, distribution of MPs in various types was significant at all the four sites. The possible factors for fibers, sheets, and fragments could be the use of plastic mulches, plastic tunnels, compost fertilizer, wastewater, dumping sites, and nearby slums.

Majority of the MPs (95%) found in the current study were less than 2 mm size and 85% were less than 1 mm in size. The distribution of MPs in various sizes and the differences in size distribution of MPs across the four sites were statistically significant. The current pattern of size fraction of MPs is similar to a pattern fractions of MPs recorded in agricultural lands in China, where 84.4% of total MPs were ≥ 1 mm and only 5.9% of MPs were 2–5 mm in size [56]. In terrestrial environment like agricultural lands, sporadic exposure to sunlight, humidity and fluctuating temperature round the year in Pakistan seems to have a role in further disintegration of MPs into smaller sized fraction. Such situation are lacking in marine environment. Implications of abundance of lower sized MPs in agricultural soils are extensive on agricultural ecosystem. Many studies have reported the altered normal flora and reduced microbial diversity and biomass in the presence of MPs in the rhizosphere [58–61].

A variety of nine different colors seems to have originated from a range of sources and diverse types of plastic materials in the soil or nearby areas. The variety also suggests the multiple pathways of contamination in addition to agricultural practices like wind transport, direct disposal, runoff water etc. Predominance of transparent and smaller sized MPs also indicate that MPs are present in the agricultural soils for a significant period of time. Intermittent exposure to sunlight, temperature and humidity in agricultural fields may cause bleaching of the colors and disintegration of the MPs into smaller sized particles. This is further supported by the fact that in the current study only transparent MPs were found in the smallest size range fraction of 0.0625–0.125 mm. Diversity of pathways and sources of MPs pollution were not only indicated by the color diversity, but also complimented by diversity of composition types of MPs in the current study. The inverse relationship between number of some color MPs and size during regression analysis indicates interesting dynamics of color and size distribution of MPs in the current study. It reflects the possible relative fragmentation pattern among various colored MPs. Some colors including transparent, green, brown, red, and blue MPs have more inclination to disintegrate into smaller particles than others. The underlying variables that render some colored MPs to disintegrate more than others need to be investigated.

In the current study about 97.2% of MPs were less than 2 mm and 85.6% were less than 1.0 mm. MPs with lower sizes has higher surface area for the adsorption of toxic chemicals like heavy metals (Al, As, Cd, Cr, Sr, Co, Cu, Fe, Mn, Ni, Pb and Zn), POPs, hydrocarbons (DDTs and PCBs), pharmaceutical products, halogens, PAHs and others [62]. These adsorbed chemicals and other desorbed additives that are added during plastic production, can cause severe damages in the ecosystem, they are present.

Despite composition analysis of fragments of more than 2mm size, a good diversity of six polymer types including Polyethylene (PE), Polyethylene terephthalate (PET), Polypropylene (PP), Polystyrene (PS), Polycarbonate (PC), and Polyvinyl Chloride (PVC) during the current study indicate diversity of sources of contamination. Possible sources of these polymers in the present study are given in Table 6. PE (52%), PET (18.7%) and PP (14.7%) are widely used and produced plastic types, hence most dominant type of polymer in the current case also. PET has widespread use in as packaging material and containers, while PET can be linked to single use plastic products. PP is commonly used in automotive parts, packaging and textile. These polymers have a range of additives and chemical that contaminate the environment throughout their persistent life. Each of the identified polymer type is reported to be absorbent of various contaminants including heavy metals [63–67]. These recorded types are also reported to adsorb a wide range of pesticides and may contaminate the agricultural ecosystems synergistically [59,68–71].

**Table 6:** Possible sources of MPs polymer types in the peri-urban agricultural lands of Lahore, Pakistan.

#	Polymer Type	Uses / possible sources
**1**	Polyethylene (PE)	Agricultural mulch, plastic tunnels; pipes, toys, bottles, garbage bags, and shopping bags in the domestic waste, compost and wastewater use, manure
**2**	Polyethylene terephthalate (PET)	Disposable items, packaging for food, fruit and drinks, construction material dumping in neighboring agricultural lands; water bottles, compost, manure
**3**	Polypropylene (PP)	Bumpers, rope, bottle caps
**4**	Polystyrene (PS)	Utensils, containers, disposable plates etc
**5**	Polycarbonate (PC)	Tunnel sheets, road dust automobile parts fragments
**6**	Polyvinyl Chloride (PVC)	Containers, pipes, cable and wire insulation, construction waste containing PVC plumbing waste.

Although there are no threshold values available to compare current contamination levels of MPs in peri-urban soil of Lahore, their diversity, types, and lower size range in ample quantities may result in further deterioration of agricultural soils. Agricultural soils around Lahore are using sewage water and organic fertilizers in routine and are already contaminated with heavy metals and pesticides. Being near to a large city with 14 million of population, these soils are also increasingly receiving MPs. It will only aggravate the already deteriorating situation, and there is a need to investigate the combined effects of all these contaminations on agricultural soils and crop production.

## Conclusions

A significant level of MPs contamination was present in the peri-urban agricultural lands of Lahore, which is greater than in many parts of the world. Peri-urban agricultural lands of Lahore are nearly uniformly polluted by microplastics. Diversity of sizes, colors, types and chemical composition of MPs indicates the diversity of contamination pathways. Current agricultural practices (use of plastic materials, wastewater irrigation, use of compost and organic fertilizers) and dumping of solid waste near agricultural lands seem possible sources of MPs. Further research is needed to focus on the relative contribution of point sources and input pathways of MPs in agricultural soils.

## References

[1] Shams M, Alam I, Mahbub MS. Plastic pollution during COVID-19: Plastic waste directives and its long-term impact on the environment. Environmental advances. 2021 Oct 1;5:100–119. doi: 10.1016/j.envadv.2021.100119 34604829 PMC8464355

[2] Cucci C, Bartolozzi G, Marchiafava V, Picollo M, Richardson E. Study of semi-synthetic plastic objects of historic interest using non-invasive total reflectance FT-IR. Microchemical Journal. 2016 Jan 1;124:889–97.

[3] Hossain S, Rahman MA, Chowdhury MA, Mohonta SK. Plastic pollution in Bangladesh: A review on current status emphasizing the impacts on environment and public health. Environmental Engineering Research. 2021 Dec;26(6).

[4] Geyer R, Jambeck JR, Law KL. 2017. Production, use, and fate of all plastics ever made. Science Advances. 2017;3(7): e1700782. 28776036 10.1126/sciadv.1700782 PMC5517107

[5] Facts PE. An analysis of European plastics production, demand and waste data. Plastics Europe. 2019.

[6] Al-Dailami A, Ahmad I, Kamyab H, Abdullah N, Koji I, Ashokkumar V, et al. Sustainable solid waste management in Yemen: environmental, social aspects, and challenges. Biomass Conversion and Biorefinery. 2022 Jun;10:1–27.

[7] Rai M, Pant G, Pant K, Aloo BN, Kumar G, Singh HB, et al. Microplastic Pollution in Terrestrial Ecosystems and Its Interaction with Other Soil Pollutants: A Potential Threat to Soil Ecosystem Sustainability. Resources. 2023 May 27;12(6):67.

[8] Fotopoulou KN, Karapanagioti HK. Degradation of Various Plastics in the Environment. Hazardous chemicals associated with plastics in the marine environment. 2019;71–92.

[9] Kunz A, Walther BA, Lowemark L, Lee YC. Distribution and quantity of microplastic on sandy beaches along the northern coast of Taiwan. Marine Pollution Bulletin. 2016;111(1–2): 126–135. doi: 10.1016/j.marpolbul.2016.07.022 27449830

[10] Blair RM, Waldron S, Phoenix VR, Gauchotte-Lindsay C. Microscopy and elemental analysis characterisation of microplastics in sediment of a freshwater urban river in Scotland, UK. Environmental Science and Pollution Research. 2019 Apr 1;26:12491–504. doi: 10.1007/s11356-019-04678-1 30848429 PMC6476856

[11] Wang C, Zhao J, Xing B. Environmental source, fate, and toxicity of microplastics. Journal of hazardous materials. 2021;407: 124357. doi: 10.1016/j.jhazmat.2020.124357 33158648

[12] Nayeri D, Mousavi SA, Almasi A, Asadi A. Microplastic abundance, distribution, and characterization in freshwater sediments in Iran: a case study in Kermanshah city. Environmental Science and Pollution Research. 2023 Apr;30(17):49817–28. doi: 10.1007/s11356-023-25620-6 36781678

[13] Zhang Q, He Y, Cheng R, Li Q, Qian Z, Lin X. Recent advances in toxicological research and potential health impact of microplastics and nanoplastics in vivo. Environmental Science and Pollution Research. 2022 Jun;29(27):40415–48. doi: 10.1007/s11356-022-19745-3 35347608

[14] Cao X., Liang Y., Jiang J., Mo A., He D. Organic additives in agricultural plastics and their impacts on soil ecosystems: Compared with conventional and biodegradable plastics. TrAC Trends in Analytical Chemistry.2023;117212.

[15] Samadi A, Kim Y, Lee SA, Kim YJ, Esterhuizen M. Review on the ecotoxicological impacts of plastic pollution on the freshwater invertebrate Daphnia. Environmental Toxicology. 2022 Nov;37(11):2615–38. doi: 10.1002/tox.23623 35907204 PMC9796382

[16] Okoye CO, Addey CI, Oderinde O, Okoro JO, Uwamungu JY, Ikechukwu CKet al. Toxic chemicals and persistent organic pollutants associated with micro-and nanoplastics pollution. Chemical Engineering Journal Advances. 2022 Apr 29;100310.

[17] Mato Y, Isobe T, Takada H, Kanehiro H, Ohtake C, Kaminuma T. Plastic resin pellets as a transport medium for toxic chemicals in the marine environment. Environtal Science & Technology. 2001;35(2):318–324. doi: 10.1021/es0010498 11347604

[18] Teuten EL, Rowland SJ, Galloway TS, Thompson RC. Thompson Potential for plastics to transport hydrophobic contaminants. Environtal Science & Technology. 2007;41(22);7759–7764. 10.1021/es071737s 18075085

[19] Campanale C, Galafassi S, Savino I, Massarelli C, Ancona V, Volta P et al. Microplastics pollution in the terrestrial environments: Poorly known diffuse sources and implications for plants. Science of the Total Environment. 2022 Jan 20;805:150431. doi: 10.1016/j.scitotenv.2021.150431 34818779

[20] Tian L, Jinjin C, Ji R, Ma Y, Yu X. Microplastics in agricultural soils: sources, effects, and their fate. Current Opinion in Environal Science & Health. 2022;25:100311.

[21] Kumar M, Xiong X, He M, Tsang DC, Gupta J, Khan E. Microplastics as pollutants in agricultural soils. Environmental Pollution. 2020 Oct 1;265:114980. doi: 10.1016/j.envpol.2020.114980 32544663

[22] De Bhowmick G, Sarmah AK. Microplastics contamination associated with land-application of biosolids–A Perspective. Current Opinion in Environmental Science & Health. 2022 Apr 1;26:100342.

[23] Sáez JA, Pedraza Torres AM, Blesa Marco ZE, Andreu-Rodríguez FJ, Marhuenda-Egea FC, Martínez-Sabater E, et al. The Effects of Agricultural Plastic Waste on theVermicompost Process and Health Status of Eisenia fetida. Agronomy. 2022 Oct 18;12(10):2547.

[24] Wiesner YK, Müller A, Bannick CG, Bednarz M, Braun U. Pathways of microplastics in soils-Detection of microplastic contents in compost using a thermal decomposition method. InEGU General Assembly Conference Abstracts 2020 May (p 22456).

[25] Jin T, Tang J, Lyu H, Wang L, Gillmore AB, Schaeffer SM. Activities of microplastics (MPs) in agricultural soil: a review of MPs pollution from the perspective of agricultural ecosystems. Journal of Agricultural and Food Chemistry. 2022 Apr 5;70(14):4182–201. doi: 10.1021/acs.jafc.1c07849 35380817

[26] Yang L, Zhang Y, Kang S, Wang Z, Wu C. Microplastics in soil: A review on methods, occurrence, sources, and potential risk. Science of the Total Environment. 2021 Aug 1;780:146546. doi: 10.1016/j.scitotenv.2021.146546 33770602

[27] Lima JZ, Cassaro R, Ogura AP, Vianna MM. A systematic review of the effects of microplastics and nanoplastics on the soil-plant system. Sustainable Production and Consumption. 2023 Apr 25.

[28] Khalid N, Aqeel M, Noman A. Microplastics could be a threat to plants in terrestrial systems directly or indirectly. Environmental Pollution. 2020 Dec 1;267:115653. doi: 10.1016/j.envpol.2020.115653 33254725

[29] Wan Y, Wu C, Xue Q, Hui X. Effects of plastic contamination on water evaporation and desiccation cracking in soil. Science of the Total Environment. 2019 Mar 1;654: 576–582. doi: 10.1016/j.scitotenv.2018.11.123 30447596

[30] Wang F, Zhang X, Zhang S, Zhang S, Sun Y. Interactions of microplastics and cadmium on plant growth and arbuscular mycorrhizal fungal communities in an agricultural soil. Chemosphere. 2020 Sep 1;254: 126791. doi: 10.1016/j.chemosphere.2020.126791 32320834

[31] Wang J., Zhang X., Li X., & Wang Z. Exposure pathways, environmental processes and risks of micro (nano) plastics to crops and feasible control strategies in agricultural regions. Journal of Hazardous Materials2023;10:132269. doi: 10.1016/j.jhazmat.2023.132269 37607458

[32] Pakistan Bureau of Statistics. 7^th^ Population and Housing Census, 2023, the Digital Census of Pakistan 2023.

[33] Hameed MU., Wahab AA., Raza HA, Haroon M, Adeel M, Rebi A, et al. A study of the imperatives and repercussions of sewage water in vegetables production in district Faisalabad Pakistan: a farmer’s perspective. Annals of the Romanian Society for Cell Biology. 2021 Nov 17;25(7):1350–1365.

[34] Masura J, Baker J, Foster G, Arthur C. Laboratory Methods for the Analysis of Microplastics in the Marine Environment: Recommendations for quantifying synthetic particles in waters and sediments. 2015.

[35] Kundu MN, Hans C, Lugomela KG. Analysis of macro- and microplastics in riverine, riverbanks, and irrigated farms in Arusha, Tanzania. Archives of Environmental Contamination and Toxicology. 2021 Jan;82(1):142–157. doi: 10.1007/s00244-021-00897-1 34741639

[36] Ragoobur D, Huerta-Lwanga E, Somaroo GD. Microplastics in agricultural soils, wastewater effluents and sewage sludge in Mauritius. Science of the Total Environment. 2021 Dec 1;798:149326. doi: 10.1016/j.scitotenv.2021.149326 34340075

[37] Boughattas I, Hattab S, Zitouni N, Mkhinini M, Missawi O, Bousserrhine N, et al. Assessing the presence of microplastic particles in Tunisian agriculture soils and their potential toxicity effects using eisenia andrei as bioindicator. Science of the Total Environment. 2021;796:148959. doi: 10.1016/j.scitotenv.2021.148959 34265609

[38] Corradini F, Meza P, Eguiluz R, Casado F, Huerta-Lwanga E, Geissen V. Evidence of microplastics accumulation in agricultural soils from sewage sludge disposal. Science of the Total Environment. 2019;671:411–420. 30933797 10.1016/j.scitotenv.2019.03.368

[39] Crossman J, Hurley RR, Futter M, Nizzetto L. Transfer and transport of microplastics from biosolids to agricultural soils and the wider environment. Science of the Total Environment. 2020;724:138334. doi: 10.1016/j.scitotenv.2020.138334 32408466

[40] Saadu I, Farsang A. Greenhouse farming as a source of macroplastic and microplastics contamination in agricultural soils: a case study from Southeast Hungary. Agrochemistry and Soil Science. 2022;71(1):43–57. 10.1556/0088.2022.00120

[41] Li S, Ding F, Flury M, Wang Z, Xu L, Li S. Macroand microplastic accumulation in soil after 32 years of plastic film mulching, China. Environmental Pollution. 2022;300:118945. 35122919 10.1016/j.envpol.2022.118945

[42] Zhang Q, Zhao M, Meng F, Xiao Y, Dai W, Luan Y. Effect of polystyrene microplastics on rice seed germination and antioxidant enzyme activity. Toxics. 2021;9(8):179. doi: 10.3390/toxics9080179 34437497 PMC8402430

[43] Yang M, Huang D, Tian Y, Zhu Q, Zhang Q, Zhu H. Influences of different source microplastics with different particle sizes and application rates on soil properties and growth of Chinese cabbage (*Brassica Chinensis* L.). Ecotoxicology and Environmental Safety. 2021 Oct 1;222:112480. 34217116 10.1016/j.ecoenv.2021.112480

[44] Choi YR, Kim Y, Yoon J, Dickson N, Kim K. Plastic contamination of forest, urban, and agricultural soils: a case study of Yeoju in the republic of Korea. Journal of Soils Sediments. 2020;21:1962–1973.

[45] Isari EA, Papaioannou D, Ioannis K, Kalavrouziotis IK, Karapanagioti HK. Microplastics in agricultural soils: a case study in cultivation of watermelons and canning tomatoes. Water. 2021;13(16):2168. 10.3390/w13162168

[46] Schothorst B, Beriot N, Lwanga EH, Geissen V. Sources of light density microplastic related to two agricultural practices: the use of compost and plastic mulch. Environments. 2021;8(4):36

[47] Corradini F, Meza P, Eguiluz R, Casado F, Huerta-Lwanga E, Geissen V. Evidence of microplastics accumulation in agricultural soils from sewage sludge disposal. Science of the total environment. 2019;671:411–420. 30933797 10.1016/j.scitotenv.2019.03.368

[48] Tagg AS, Brandes E, Fischer F, Fischer D, Brandt J, Labrenz M. Agricultural application of microplastic-rich sewage sludge leads to further uncontrolled contamination. Science of the Total Environment. 2022 Feb 1;806:150611. doi: 10.1016/j.scitotenv.2021.150611 34610398

[49] Katsumi N, Kusube T, Nagao S, Okochi H. Accumulation of microcapsules derived from coated fertilizer in paddy fields. Chemosphere. 2020;267:129185. doi: 10.1016/j.chemosphere.2020.129185 33352372

[50] Bigalke M, Fieber M, Foetisch A, Reynes J, Tollan P. Microplastics in agricultural drainage water: a link between terrestrial and aquatic microplastic pollution. Science of the Total Environment. 2022;806:150709. doi: 10.1016/j.scitotenv.2021.150709 34600992

[51] Fakour H, Lo SL, Yoashi NT, Massao AM, Lema NN, Mkhontfo FB, Et al. Quantification and analysis of microplastics in farmland soils: characterization, characterization, sources, and pathways. Agriculture. 2021 Apr 8;11(4):330.

[52] Liu M, Lu S, Song Y, Lei L, Hu J, Lv W, et al. Microplastic and mesoplastic pollution in farmland soils in suburbs of Shanghai, China. Environmental Pollution. 2018;242: 855–862. doi: 10.1016/j.envpol.2018.07.051 30036839

[53] Zhang GS, Liu YF. The distribution of microplastics in soil aggregate fractions in southwestern China. Science of the Total Environment. 2018;642: 12–20. doi: 10.1016/j.scitotenv.2018.06.004 29894871

[54] Ding L, Zhang S, Wang X, Yang X, Zhang C, Qi Y, et al. The occurrence and distribution characteristics of microplastics in the agricultural soils of Shaanxi Province, in north-western China. Science of the Total Environment. 2020;720: 137525. doi: 10.1016/j.scitotenv.2020.137525 32325574

[55] Rafique A, Irfan M, Mumtaz M, Qadir A,. Spatial distribution of microplastics in soil with context to human activities: a case study from the urban center. Environmental Monitoring and Assessment. 2020 Nov;192:1–3. doi: 10.1007/s10661-020-08641-3 33009943

[56] Yu L, Zhang J, Liu Y, Chen L, Tao S, Liu W. Distribution characteristics of microplastics in agricultural soils from the largest vegetable production base in China. Scince of the Total Environment. 2021;756: 143860. doi: 10.1016/j.scitotenv.2020.143860 33302081

[57] Lahore Chamber of Commerce and Industry. Textile industry in Lahore, Pakistan. Accessed on 01-09-2023 at https://lcci.com.pk/rptdirectory2.php?sector=01&busines_type=1

[58] Muroi F, Tachibana Y, Kobayashi Y, Sakurai T, Kasuya K. Influences of poly (butylene adipate-co-terephthalate) on soil microbiota and plant growth. Polymer Degradation and Stability. 2016;129:338–46.

[59] Wang J, Lv S, Zhang M, Chen G, Zhu T, Zhang S, et al. Effects of plastic film residues on occurrence of phthalates and microbial activity in soils. Chemosphere. 2016;151:171–7. doi: 10.1016/j.chemosphere.2016.02.076 26938679

[60] Awet TT, Kohl Y, Meier F, Straskraba S, Grün AL, Ruf T, et al. Effects of polystyrene nanoparticles on the microbiota and functional diversity of enzymes in soil. Environmental Sciences Europe. 2018;30:1–10. 10.1186/s12302-018-0140-6. 29963347 PMC5937892

[61] Ren X, Tang J, Liu X, Liu Q. Effects of microplastics on greenhouse gas emissions and the microbial community in fertilized soil. Environmental Pollution. 2020 Jan 1;256:113347. doi: 10.1016/j.envpol.2019.113347 31672352

[62] Verla AW, Enyoh CE, Verla EN, Nwarnorh KO. Microplastic–toxic chemical interaction: a review study on quantified levels, mechanism and implication. SN Applied Science. 2019;1(11): 1–30.

[63] Dong Y, Gao M, Song Z, Qiu W. Adsorption mechanism of As (III) on polytetrafluoroethylene particles of different size. Environmental Pollution. 2019;254: 112950. doi: 10.1016/j.envpol.2019.07.118 31394339

[64] Zhang J, Hamza A, Xie Z, Hussain S, Brestic M, Tahir MA, et al. Arsenic transport and interaction with plant metabolism: Clues for improving agricultural productivity and food safety. Environmental Pollution. 2021;290: 117987. doi: 10.1016/j.envpol.2021.117987 34425370

[65] Godoy V, Blázquez G, Calero M, Quesada L, Martín-Lara M. The potential of microplastics as carriers of metals. Environmental Pollution. 2019;255: 113363. doi: 10.1016/j.envpol.2019.113363 31614247

[66] Collin S, Baskar A, Geevarghese DM, Ali MNVS, Bahubali P, Choudhary R, et al. Bioaccumulation of Lead (Pb) and its effects in plants: A review. Journal of Hazardous Materials Letters. 2022 Jul 20:100064.

[67] El Rasafi T, Oukarroum A, Haddioui A, Song H, Kwon EE, Bolan N, et al. Cadmium stress in plants: A critical review of the effects, mechanisms, and tolerance strategies. Critical Reviews in Environmental Science and Technology. 2022;52(5): 675–726.

[68] Wang T, Yu C, Chu Q, Wang F, Lan T, Wang J. Adsorption behavior and mechanism of five pesticides on microplastics from agricultural polyethylene films. Chemosphere. 2020;244: 125491. doi: 10.1016/j.chemosphere.2019.125491 31835051

[69] Sunta U, Prosenc F, Trebše P, Bulc TG, Kralj MB. Adsorption of acetamiprid, chlorantraniliprole and flubendiamide on different type of microplastics present in alluvial soil. Chemosphere. 2020;261: 127762. doi: 10.1016/j.chemosphere.2020.127762 32738715

[70] Zhang C, Lei Y, Qian J, Qiao Y, Liu J, Li S, et al. Sorption of organochlorine pesticides on polyethylene microplastics in soil suspension. Ecotoxicology and Environmental Safety. 2021;223: 112591. doi: 10.1016/j.ecoenv.2021.112591 34364123

[71] Sun W, Meng Z, Li R, Zhang R, Jia M, Yan S, et al. Joint effects of microplastic and dufulin on bioaccumulation, oxidative stress and metabolic profile of the earthworm (Eisenia fetida). Chemosphere. 2021;263: 128171. doi: 10.1016/j.chemosphere.2020.128171 33297140

